# Quantitative Evaluation for Magnetoelectric Sensor Systems in Biomagnetic Diagnostics

**DOI:** 10.3390/s22031018

**Published:** 2022-01-28

**Authors:** Eric Elzenheimer, Christin Bald, Erik Engelhardt, Johannes Hoffmann, Patrick Hayes, Johan Arbustini, Andreas Bahr, Eckhard Quandt, Michael Höft, Gerhard Schmidt

**Affiliations:** 1 Digital Signal Processing and System Theory, Institute of Electrical Engineering and Information Technology, Faculty of Engineering, Kiel University, Kaiserstr. 2, 24143 Kiel, Germany; ee@tf.uni-kiel.de (E.E.); cbal@tf.uni-kiel.de (C.B.); eren@tf.uni-kiel.de (E.E.); jph@tf.uni-kiel.de (J.H.); 2 Inorganic Functional Materials, Institute for Materials Science, Faculty of Engineering, Kiel University, Kaiserstr. 2, 24143 Kiel, Germany; pah@tf.uni-kiel.de (P.H.); eq@tf.uni-kiel.de (E.Q.); 3 Sensor System Electronics, Institute of Electrical Engineering and Information Technology, Faculty of Engineering, Kiel University, Kaiserstr. 2, 24143 Kiel, Germany; jrsa@tf.uni-kiel.de (J.A.); ab@tf.uni-kiel.de (A.B.); 4 Microwave Engineering, Institute of Electrical Engineering and Information Technology, Faculty of Engineering, Kiel University, Kaiserstr. 2, 24143 Kiel, Germany; mh@tf.uni-kiel.de

**Keywords:** application specific signal evaluation, magnetoelectric sensors, quantitative sensor system characterization, sensor system performance

## Abstract

Dedicated research is currently being conducted on novel thin film magnetoelectric (ME) sensor concepts for medical applications. These concepts enable a contactless magnetic signal acquisition in the presence of large interference fields such as the magnetic field of the Earth and are operational at room temperature. As more and more different ME sensor concepts are accessible to medical applications, the need for comparative quality metrics significantly arises. For a medical application, both the specification of the sensor itself and the specification of the readout scheme must be considered. Therefore, from a medical user’s perspective, a system consideration is better suited to specific quantitative measures that consider the sensor readout scheme as well. The corresponding sensor system evaluation should be performed in reproducible measurement conditions (e.g., magnetically, electrically and acoustically shielded environment). Within this contribution, an ME sensor system evaluation scheme will be described and discussed. The quantitative measures will be determined exemplarily for two ME sensors: a resonant ME sensor and an electrically modulated ME sensor. In addition, an application-related signal evaluation scheme will be introduced and exemplified for cardiovascular application. The utilized prototype signal is based on a magnetocardiogram (MCG), which was recorded with a superconducting quantum-interference device. As a potential figure of merit for a quantitative signal assessment, an application specific capacity (ASC) is introduced. In conclusion, this contribution highlights metrics for the quantitative characterization of ME sensor systems and their resulting output signals in biomagnetism. Finally, different ASC values and signal-to-noise ratios (SNRs) could be clearly presented for the resonant ME sensor (SNR: −90 dB, ASC: $9.8\times 10^{-7}$ dB Hz) and also the electrically modulated ME sensor (SNR: −11 dB, ASC: 23 dB Hz), showing that the electrically modulated ME sensor is better suited for a possible MCG application under ideal conditions. The presented approach is transferable to other magnetic sensors and applications.

## 1. Introduction

Medical diagnostics based on electrical signal acquisition methods such as electrocardiography (ECG) or electroencephalography (EEG) are an established routine in clinical practice. These methods have been researched for decades [1,2]. Nowadays, room-temperature magnetic field sensors are being investigated, such as optically pumped magnetometers [3,4], xMR sensors [5], orthogonal fluxgates [6], and many more. These sensor concepts promise several advantages and enable contactless signal acquisition by detecting the magnetic field strength or the magnetic flux density. Obtaining biomagnetic signals is beneficial compared to the standard electrical methods for several reasons. Magnetic sensing promises increased spatial resolution [7], it enables better positioning with less exogenous signal artifacts and the nearly constant relative permeability [8], which prevents physiologic signals from being changed by the elements of the body (tissue, bones, etc). In particular, the ongoing research of thin-film magnetoelectric (ME) sensors enables new areas of signal acquisition in medicine since they do not require cryogenic cooling or thermal heating for sensor operation [9,10,11]. These sensors are easy to use, provide unprecedented flexibility and are operational in the presence of interference fields such as the Earth’s magnetic field [12,13]. Magnetic recording techniques have the potential to support and replace traditional electrode-based (electrical) methods by default [14]. The performance of a magnetic field sensor is usually described by its sensor-specific properties, e.g., operation temperature, inherent noise, dynamic range (in the sense of amplitude range of operation), bandwidth and sensitivity [15], as exemplified for two current biomagnetic ME sensor types in Table 1.

**Figure 1:**
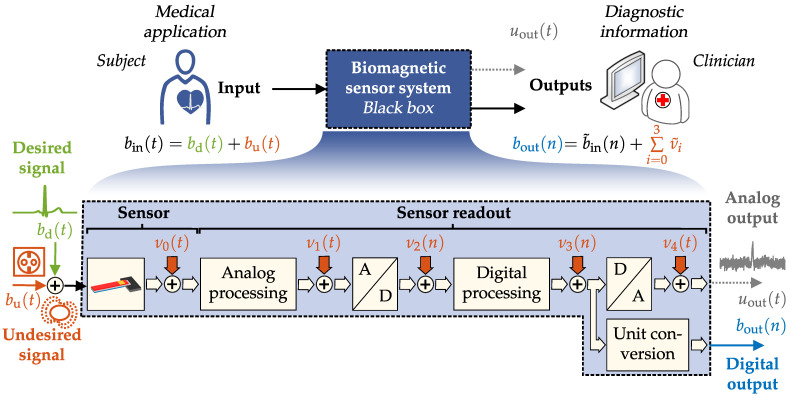
Schematic representation of a typical biomagnetic sensor system.

For medical applications, it is not sufficient to consider only the sensing element specification because the overall performance of a sensor system is a superposition of all its subsystems and their individual performances. This includes especially the sensor readout electronics. Since the application of magnetic sensors is a relatively new field of research, often only the sensing element’s specifications are provided. The specification of the entire sensor system must be taken into consideration for determining if a sensor is appropriate for a specific application. To exemplify, in a medical applications the question could be asked, whether a signal of interest, for example, the heartbeat, could be measured for diagnostic purposes. From the viewpoint of a medical application, it does not matter where potential disturbances originate. Therefore, a sensor system in a biomagnetic application can be considered a black box. This black box is evaluated with its corresponding system metrics. A simplified representation of such an approach is shown in Figure 1.

In this contribution, magnetic field signals created by physiological means are considered the input signals of interest $b_{d}\left(t\right)$ (desired input signals). This is exemplified by the signal generated from the human heart. The system input $b_{\mathrm{in}}\left(t\right)$ consists of an additive undesired magnetic signal $b_{u}\left(t\right)$ from environmental disturbances (coexisting magnetic fields). The available field at the system input can be converted with a magnetic field sensor into a proportional measurand, typically a time-dependent voltage. The sensor signal is read out in analog form within the sensor system, digitally processed, and provided as a signal at the output. The output can also be taken in form of a sample dependent field strength $b_{\mathrm{out}}\left(n\right)$ after unit conversion (voltage → magnetic flux density) or analog as time-dependent voltage $u_{\mathrm{out}}\left(t\right)$. In the overall system, each process step has an individual transfer or conversion function and noise characteristic. At the digital output $b_{\mathrm{out}}\left(n\right)$, the signal can be considered as the sum of the input signal $b_{\mathrm{in}}\left(n\right)$ and a noise superposition of all involved noise components $\nu_{0},\ldots ,\nu_{3}$. The noise at the system output is a superposition of different uncorrelated random processes [17]. For an application, it is not decisive from where noise contributions originate. As a consequence, the noise power spectral densities or, respectively, the noise amplitude densities superimpose [18]. Finally, this view allows a quantitative description of a sensor system from a user perspective and permits comparing sensor systems for a specific medical discipline or new biomagnetic applications. Since diagnostic information depends mainly on signal characteristics, an application-specific signal evaluation scheme will be presented. This enables an improved quantitative description of the system’s suitability. In summary, this contribution highlights metrics for magnetic sensor systems and offers an application-oriented signal evaluation scheme for biomagnetic applications. The remainder of this contribution is organized as follows: In Section 2, different metrics for sensor system evaluation will be introduced. Since diagnostic information depends mainly on signal characteristics, figures of merit for signal evaluation will be supplementary defined in Section 3. Then, in Section 4, an exemplary evaluation will be executed for two different ME sensor systems: a exchange bias magnetoelectric sensor and an electrically modulated ME sensor. Based on these findings, a signal evaluation will be performed, exemplified by a cardiovascular application in Section 4.3. Finally, in Section 5 the individual results will be discussed.

## 2. Evaluation Metrics for Magnetoelectric Sensor Systems

Quantitative evaluation metrics are of importance for the characterization and comparison of biomagnetic sensor systems. Key metrics are the *Input–Output–Amplitude–Relation* and *frequency response* [19]. First, the Input–Output–Amplitude–Relation will be discussed, since no explicit system knowledge and presumptions are required.

### 2.1. Input–Output–Amplitude–Relation

**Figure 2:**
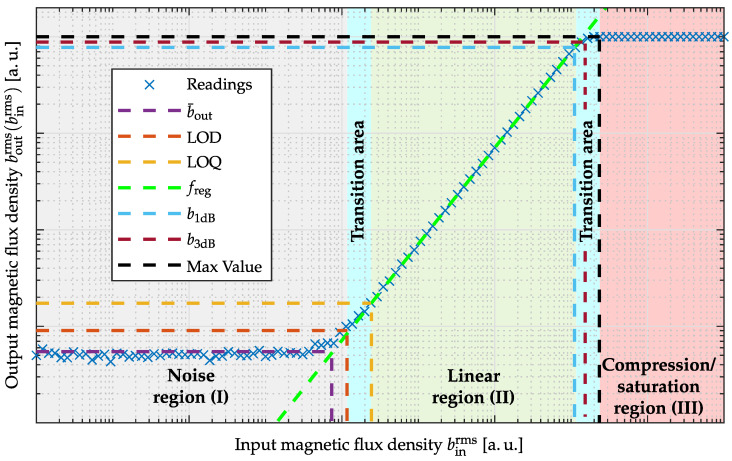
Input–Output–Amplitude–Relation with labeling of the typical regions (noise region (I), linear region (II) and compression/saturation region (III)). Transition areas are marked in cyan. Furthermore, characteristic quantities, mean value within noise region, *Limit-of-Detection* (LOD), *Limit-of-Quantification* (LOQ), *1-dB-compression-point*, *3-dB-compression-point* and *maximum value* are marked in different colors.

A typical *Input–Output–Amplitude–Relation* of a sensor system is illustrated in Figure 2. It can be divided into three different regions. In the first region (I; gray shaded area), the magnetic signal is so small that the system noise dominates at the output. If the magnetic signal is large enough and exceeds the system noise, the system output increases linearly with the input amplitude (II; green shaded area) until it is limited by compression and saturation effects (III; red shaded area). Limiting factors can be the sensor’s dynamic range or the readout electronics characteristics and limitations, e.g., operating voltages, sensor’s dynamic range (DR). In addition, transition areas can be identified (cyan shaded area) which cannot be unambiguously assigned to one of the areas mentioned above. For a quantification of the Input–Output–Amplitude–Relation at a particular excitation frequency (typically 10 Hz, 1 kHz, or resonance frequency), P∈N pairs of root-mean-square (RMS) input and output values are required.

(1) $$b_{\mathrm{in}}^{\mathrm{rms}}={\left[b_{\mathrm{in}}^{\mathrm{rms}}\left(0\right),\;\;\;b_{\mathrm{in}}^{\mathrm{rms}}\left(1\right),\;\;\;\ldots \;\;\;,b_{\mathrm{in}}^{\mathrm{rms}}\left(P-1\right)\right]}^{T},$$

(2) $$b_{\mathrm{out}}^{\mathrm{rms}}={\left[b_{\mathrm{out}}^{\mathrm{rms}}\left(0\right),\;\;\;b_{\mathrm{out}}^{\mathrm{rms}}\left(1\right),\;\;\;\ldots \;\;\;,b_{\mathrm{out}}^{\mathrm{rms}}\left(P-1\right)\right]}^{T},$$

where $b_{\mathrm{in}}^{\mathrm{rms}}$ are the input RMS values of the sensor system and $b_{\mathrm{out}}^{\mathrm{rms}}$ are the acquired RMS output values. Since an additional DC offset has, in general, a significant influence on the curve progression including the derived quantities, it should be identified and minimized for functional determination for all system output values within $b_{\mathrm{out}}^{\mathrm{rms}}$. Characteristic quantities such as *Limit-of-Detection*, *Limit-of-Quantification*, *maximum value*, and *1-dB-compression-point* can be defined for quantitative description of the Input–Output–Amplitude–Relation.

#### 2.1.1. Limit-of-Detection

The *Limit-of-Detection* (LOD) of a biomagnetic sensor system describes the smallest measurable magnetic flux density where a magnetic field can be reliably detected [20]. For LOD estimation, the values $b_{\mathrm{out}}^{\mathrm{rms}}$, where no signal can be reliably detected and the system noise dominates, are of interest. This condition is in general fulfilled if the desired signal is less than the effective magnetic noise amplitude $b_{n}^{\mathrm{rms}}$ corresponding to

(3) $$b_{\mathrm{out}}^{\mathrm{rms}}\left(i\right)<b_{n}^{\mathrm{rms}}.$$

The LOD can be determined from K∈N measurement points of interest, where the system noise dominates [21,22]. The LOD can be estimated by the mean value $\mu_{n}$ plus three times the standard deviation $\sigma_{n}$ of the predefined measurement points with:

(4) $$LOD=\underset{{\bar{b}}_{\mathrm{out}}=\mu_{n}}{\underset{︸}{\frac{1}{K}\;\cdot \;\sum_{i=0}^{K-1}b_{\mathrm{out}}^{\mathrm{rms}}\left(i\right)}}+3\;\cdot \;\underset{\sigma_{n}}{\underset{︸}{\sqrt{\frac{\sum_{i=0}^{K-1}\left[b_{\mathrm{out}}^{\mathrm{rms}}\left(i\right)-{\bar{b}}_{\mathrm{out}}\right]2}{K-1}}}}.$$

**Figure 3:**
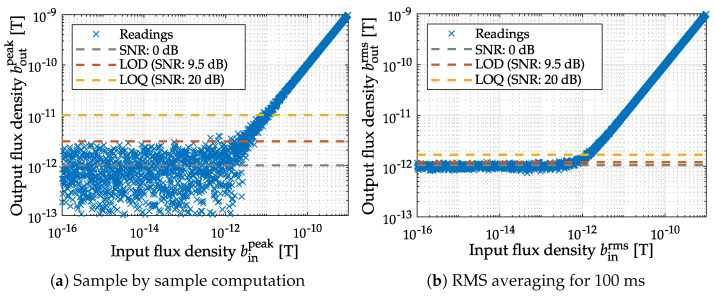
Different methods for LOD computation for the same signal. The simulated sample by sample computation (**a**) of the standard deviation and the mean value yields the same results as the stochastic parameters of the applied random process ($\mu_{n}\;=\;0$, $\sigma_{n}\;=\;1\;\mathrm{pT}$). The averaging window applied in (**b**) results in a reduced spread and therefore an SNR gain at the cost of a reduction in bandwidth ($\mu_{n}\;=\;1\;\mathrm{pT}$, $\sigma_{n}\;=\;70\;\mathrm{fT}$).

The LOD serves as a criterion of reliable evidence and is provided in magnetic sensor systems as an RMS value with the unit T for a particular excitation frequency. It defines the lowest magnetic field that the sensor system can reliably detect [19]. A spectral LOD consideration in T/$\sqrt{\mathrm{Hz}}$ is occasionally used instead, especially for modulated magnetic sensors. The amplitude density with unit T/$\sqrt{\mathrm{Hz}}$ is related to the RMS value with unit T by Parseval’s theorem [23,24]. In general, the LOD value relies on the applied measurement procedure, as illustrated in Figure 3 and has to be defined in detail. Implicit filtering of the output signal by averaging methods [11] will result in very optimistic LOD determinations, which are only realizable in applications with equal bandwidth requirements. Consequently, the LOD will only be reproducible in an experimental setup with identically chosen parameters. Therefore, additional applied filters (lowpass, highpass, bandpass filters) should be specified by their cutoff frequencies. As an assessing bandwidth, the supported frequency range of the sensing element should be chosen. In addition, the window length for RMS amplitude calculation must be stated, whereby one period of the magnetic excitation signal should be used. The resulting RMS value corresponds to the standard deviation of a noise process with zero mean. Since the calculation can also be performed in the frequency domain, the RMS amplitude can be estimated by determining the square root out of the sum from power spectral density (PSD) values. The obtainable results are equivalent [23,24].

#### 2.1.2. Limit-of-Quantification

Another quantity in connection with the detection limit is the *Limit-of-Quantification* (LOQ), which defines the boundary from which a measured value can be reliably quantified [21,22]. The LOQ can be expressed as follows:

(5) $$LOQ={\bar{b}}_{\mathrm{out}}+10\;\cdot \;\sqrt{\frac{\sum_{i=0}^{K-1}{\left[b_{\mathrm{out}}^{\mathrm{rms}}\left(i\right)-{\bar{b}}_{\mathrm{out}}\right]}^{2}}{K-1}}.$$

Compared to the previously defined LOD (see Equation (4)) the required standard deviation is set by a factor of 10/3 higher [21,22]. This corresponds to a signal-to-noise ratio (SNR) of 20 dB, ensuring that signal amplitudes above LOQ can be detected at the system output in the time domain.

#### 2.1.3. Linear Region

The linear region given by the Input–Output–Amplitude–Relation (Figure 2) can be described using a linear function approximation [19]. For this purpose, an affine linear regression $f_{\mathrm{reg}}\left(\;\cdot \;\right)$ can be performed using the measurement values $b_{\mathrm{out}}^{\mathrm{rms}}$ as a function of the excitation signal amplitudes $b_{\mathrm{in}}^{\mathrm{rms}}$ given by

(6) $$b_{\mathrm{out}}^{\mathrm{rms}}\left(i\right)=f_{\mathrm{reg}}\left(b_{\mathrm{in}}^{\mathrm{rms}}\left(i\right)\right)=\alpha +\beta \;\cdot \;b_{\mathrm{in}}^{\mathrm{rms}}\left(i\right)\;\;\;\forall \;\;\;b_{\mathrm{in}}^{\mathrm{rms}}\left(i\right),\;LOQ<b_{\mathrm{out}}^{\mathrm{rms}}\left(i\right)<b_{1\mathrm{dB}}.$$

In the case of curve compression for large input amplitudes originating from system limitations based on saturation effects (nonlinearities), corresponding data points should be excluded. Therefore, signal amplitudes $b_{\mathrm{in}}^{\mathrm{rms}}$ should be bigger than LOQ and smaller than the *1-dB-Compression-Point*$b_{1\mathrm{dB}}$ (cf. Section 2.1.4). Unique solutions for α and β can be found by minimizing the sum of squared deviations as follows for the remaining *W* data points:

(7) $$\begin{array}{cc} \beta & =\frac{\sum_{i=0}^{W-1}b_{\mathrm{in}}^{\mathrm{rms}}\left(i\right)\;\cdot \;b_{\mathrm{out}}^{\mathrm{rms}}\left(i\right)-W\;\cdot \;\mu_{\mathrm{in}}\;\cdot \;\mu_{\mathrm{out}}}{\sum_{i=0}^{W-1}{\left[b_{\mathrm{in}}^{\mathrm{rms}}\left(i\right)\right]}^{2}-W\;\cdot \;{\left[\mu_{\mathrm{in}}\right]}^{2}}\;\;\;\mathrm{and}\;\;\;\alpha =\mu_{\mathrm{out}}-\beta \;\cdot \;\mu_{\mathrm{in}}\\ \mathrm{with}\;\;\;\mu_{\mathrm{in}} & =\frac{1}{W}\;\cdot \;\sum_{i=0}^{W-1}b_{\mathrm{in}}^{\mathrm{rms}}\left(i\right)\;\;\;\mathrm{and}\;\;\;\mu_{\mathrm{out}}=\frac{1}{W}\;\cdot \;\sum_{i=0}^{W-1}b_{\mathrm{out}}^{\mathrm{rms}}\left(i\right). \end{array}$$

Finally, it is required that not all values in $b_{\mathrm{in}}^{\mathrm{rms}}$ are equal, which ensures that the denominator of β is different from zero [25]. This primary requirement is fulfilled due to the performed amplitude variation at the system input.

#### 2.1.4. 1-dB-Compression-Point and 3-dB-Compression-Point

In general, sensor systems must have high linearity in their operating range to prevent unwanted signal components at the output [26]. Therefore, if a limitation of the curve progression is perceived and compression exists, the maximum system output $b_{\max}$ could be determined. For this purpose L∈Z data points are used, which lay in this specific region (compression/saturation region, see Figure 2). For identification of $b_{\max}$ the mean value of those data points can be calculated by:

(8) $$b_{\max}=\frac{1}{L}\;\cdot \;\sum_{i=0}^{L-1}b_{\mathrm{out}}^{\mathrm{rms}}\left(i\right).$$

For most standard magnetic field sensors, $b_{\max}$ is limited by the operating voltage of the readout electronics. In the case of ultra-sensitive magnetic field sensors, the system limitation results from saturation effects. Both effects have the consequence that, above a certain input level, the amplitude of the system output is limited with co-occurring non-linearities. A quantitative measure of linearity can be obtained using the *1-dB-Compression-Point* ($b_{1\mathrm{dB}}$) and the *3-dB-Compression-Point* ($b_{3\mathrm{dB}}$), which specify the input level at which the real transfer characteristic deviates from the regression function with ideal characteristic (see Equation (6)) by 1 dB or 3 dB, respectively. The $b_{1\mathrm{dB}}$ and $b_{3\mathrm{dB}}$ point can be determined as follows:

(9) $$b_{1\mathrm{dB}}=b_{\mathrm{in}}^{\mathrm{rms}}\left(i\right)\;\mathrm{with}\;20\;\cdot \;\log_{10}\left(\frac{b_{\mathrm{out}}^{\mathrm{rms}}\left(i\right)}{f_{\mathrm{reg}}\left(b_{\mathrm{in}}^{\mathrm{rms}}\left(i\right)\right)}\right)\overset{!}{=}-1\;\mathrm{dB},$$

(10) $$b_{3\mathrm{dB}}=b_{\mathrm{in}}^{\mathrm{rms}}\left(i\right)\;\mathrm{with}\;20\;\cdot \;\log_{10}\left(\frac{b_{\mathrm{out}}^{\mathrm{rms}}\left(i\right)}{f_{\mathrm{reg}}\left(b_{\mathrm{in}}^{\mathrm{rms}}\left(i\right)\right)}\right)\overset{!}{=}-3\;\mathrm{dB}.$$

#### 2.1.5. Dynamic Range

The supported *dynamic range* (DR) of the system can be specified using LOQ as the lower limit and $b_{1\mathrm{dB}}$ as the upper limit. The dynamic range is given by:

(11) $$DR=20\;\cdot \;\log_{10}\left(\frac{b_{1\mathrm{dB}}}{LOQ}\right)\mathrm{dB},$$

and is provided in dB units [15].

#### 2.1.6. Determination of the Input–Output–Amplitude–Relation

For the determination of the *Input–Output–Amplitude–Relation*, precise amplitude knowledge of the excitation signal $b_{d}\left(t\right)$ and a measurement of the output signal ($u_{\mathrm{out}}$; $b_{\mathrm{out}}$) are necessary. Therefore, a high precision A/D converter combined with a known magnetic reference field with frequency $f_{\mathrm{exc}}$ is used. The magnetic field is generated with a calibrated cylindrical coil within a magnetically shielded environment (permalloy cylinder). The calibrated coil is excited with an alternating current $i_{\mathrm{ac}}\left(i,t\right)$ at the frequency $f_{\mathrm{exc}}$ generated by an ultra-low-noise current source. The parameters $i_{\mathrm{ac}}$ and $f_{\mathrm{exc}}$ have to be chosen such that the following relation is valid:

(12) $$b_{\mathrm{in}}^{\mathrm{rms}}\left(i\right)\propto \frac{{\hat{i}}_{\mathrm{ac}}\left(i\right)}{\sqrt{2}}\;\mathrm{with}\;i_{\mathrm{ac}}\left(i,t\right)={\hat{i}}_{\mathrm{ac}}\left(i\right)\;\cdot \;\sin\left(2\;\pi \;f_{\mathrm{exc}}\;t\right).$$

The current source serves as the generator for the coil and as the reference signal. The resulting magnetic flux density should be varied linearly from zero to the maximum assessable flux density of the system. The saturation region may not be reachable for all sensor types. The sensor’s sensing area should be placed in the center of the coil. This approach enables the identification of the detection limit, the system behavior, and saturation effects through operating voltage or sensor dynamic limits. Finally, the measured RMS magnetic flux density at system output $b_{\mathrm{out}}^{\mathrm{rms}}\left(i\right)$ is plotted against the applied AC magnetic field amplitude $b_{\mathrm{in}}^{\mathrm{rms}}\left(i\right)$.

### 2.2. Frequency Response (Magnitude and Phase Response)

For the following considerations, it has to be assumed that the sensor system is a linear time-invariant (LTI) system that is analyzed in the discrete time-domain. Even though most sensor systems, which in some way rely on ferromagnetic material, do not have strictly linear behavior, it is convenient to assume that the sensor systems are at least approximately linear in their operation regime for small input signals (*small signal consideration*). Furthermore, an existing DC offset in the *Input–Output–Amplitude–Relation*, especially for $b_{\mathrm{in}}^{\mathrm{rms}}\left(i\right)=0$, must be identified and minimized. The precondition of time-invariance is not fulfilled by default because the sensor system performance varies in time due to changes in environmental conditions (e.g., Earth’s magnetic field) and changes in their internal environment (e.g., operating temperature). That being said, time-invariance can be assumed for a short period of system evaluation. In conclusion, the LTI conditions are achievable under the given assumptions, and consequently, the sensor system can be uniquely characterized by its causal real-valued impulse response *h* with N∈N sample values:

(13) $$h={\left[h(0),\;\;\;h(1),\;\;\;\ldots \;\;\;,h(N-1)\right]}^{T}.$$

**Figure 4:**
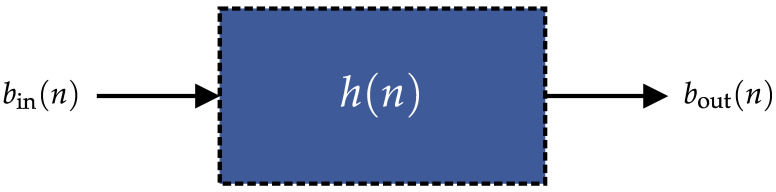
Biomagnetic LTI sensor system with impulse response h(n).

The output $b_{\mathrm{out}}\left(n\right)$ of a sensor system (see Figure 4) can be generally determined by applying a convolution with the impulse response h(n) to any input signal $b_{\mathrm{in}}\left(n\right)$ corresponding to:

(14) $$b_{\mathrm{out}}\left(n\right)=h\left(n\right)*b_{\mathrm{in}}\left(n\right)=\sum_{i=0}^{N-1}h\left(i\right)\;\cdot \;b_{\mathrm{in}}\left(n-i\right).$$

Consequently, the system signal output will be a sum of time-shifted versions of the input signal each weighted by an impulse response coefficient. The complex-valued frequency response $H(e^{j\Omega })$ is the frequency domain representation of the impulse response given by: (15)
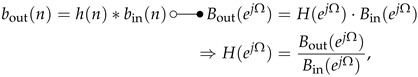

whereby symbol 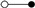 abbreviates a Fourier transform for discrete signals in the one direction and its inverse counterpart in the other. The frequency response $H(e^{j\Omega })$ of the system significantly influences the signal characteristics. Therefore, $H(e^{j\Omega })$ is of particular interest for the determination of the transfer characteristic of a sensor system, because a system impact on the magnitude and phase exists and must be considered for any application [27]. A commonly used approach for frequency response estimation can be performed by exciting the sensor system in the steady-state (transient effects are no longer present in the system) with a sinusoidal alternating magnetic field. A successive excitation with M∈N different discrete angular frequencies $\Omega_{\mu }$ with μ=0,…,M−1 in the frequency range of interest enables estimation of the absolute magnitude of $H(e^{j\Omega })$ (amplitude response) represented by

(16) $$|\hat{H}\left(e^{j\Omega_{\mu }}\right)|=\frac{\left|{\hat{B}}_{\mathrm{out}}\left(e^{j\Omega_{\mu }}\right)\right|}{\left|{\hat{B}}_{\mathrm{in}}\left(e^{j\Omega_{\mu }}\right)\right|},$$

and the corresponding phase estimation (phase response) given by:

(17) $$\hat{\Phi }\left(e^{j\Omega_{\mu }}\right)=\arg\left\{\hat{H}\left(e^{j\Omega_{\mu }}\right)\right\}=\arctan\left(\frac{ℑ\left\{\hat{H}\left(e^{j\Omega_{\mu }}\right)\right\}}{ℜ\left\{\hat{H}\left(e^{j\Omega_{\mu }}\right)\right\}}\right).$$

The amplitude response $\left|\hat{H}\left(e^{j\Omega_{\mu }}\right)\right|$ is usually presented in dB units and plotted in a double logarithmic scale [26]. The phase angle $\hat{\Phi }\left(e^{j\Omega_{\mu }}\right)$ is provided in degree units and presented in a semi-logarithmic scale. The phase information is essential since it indicates the phase change introduced by the sensor system, which is required for phase-sensitive applications, special readout schemes, and medical signal evaluations.

Subsequently, the result is influenced by choice of supporting points $\Omega_{\mu }$, which limits the accuracy for amplitude and phase response. Furthermore, an exact knowledge of the excitation signal $b_{\mathrm{in}}\left(n\right)$ and a phase-synchronous signal evaluation are essential prerequisites. This fact necessitates the use of a lock-in-amplifier. The accuracy of the system identification process can be improved if the complete frequency range of interest is excited simultaneously by a broadband signal, for example, white noise or a maximum length sequence. Based on the recorded output signal and the input signal, the transfer characteristic can then be estimated in the frequency domain [24]. Another system identification approach could be realized by determining the impulse response with a gradient-based method based on the input signal and the corresponding output signal [28]. Due to the necessity of a detailed phase response evaluation, the frequency response identification process is commonly performed with lock-in amplifiers by a successive mono-frequent excitation. Therefore, this standard method is established in current analyzers systems and has been applied successfully for years [29].

**Figure 5:**
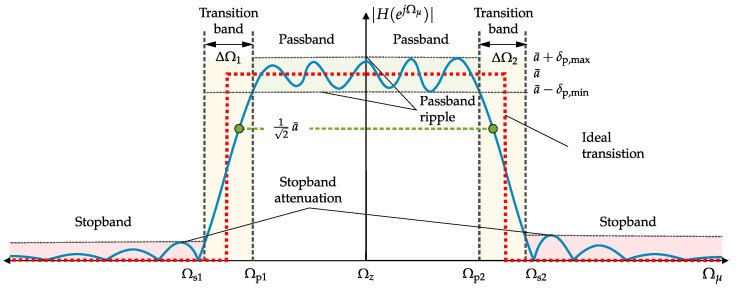
Amplitude response (asymmetrical passband) with predominant bandpass characteristic including metrics labeling. For a response with lowpass characteristic, only the right abscissa axis is required.

In general, it is helpful to describe the amplitude response of a sensor system with quantitative metrics because the magnitude behavior can be predominantly assigned to a bandpass or lowpass characteristic. A typical amplitude response of a sensor system with bandpass characteristic is shown in Figure 5. For this, the following metrics can be defined [24]:

- Mean Passband Amplitude
  (18) $$\bar{a}=\frac{1}{M}\sum_{\mu =0}^{M-1}\left|\hat{H}\left(e^{j\Omega_{\mu }}\right)\right|\;\;\mathrm{for}\;\;\Omega_{p1}\leq \Omega_{\mu }\leq \Omega_{p2}$$
- Passband Ripple
  (19) $$\begin{array}{cc} \delta_{p}=20\;\cdot \;\log_{10}\left(\frac{\bar{a}+\delta_{p,\max}}{\bar{a}-\delta_{p,\min}}\right)\;\;\; & \mathrm{with}\;\;\;\delta_{p,\max}=\max\left\{|\hat{H}\left(e^{j\Omega_{\mu }}\right)|\right\}\\ \mathrm{and}\;\;\;\delta_{p,\min}=\min\left\{|\hat{H}\left(e^{j\Omega_{\mu }}\right)|\right\}\;\; & \mathrm{for}\;\;\Omega_{p1}\leq \Omega_{\mu }\leq \Omega_{p2} \end{array}$$
- Passband Edge Frequencies
  (20) $$\Omega_{p1}=\arg\left\{\left|\hat{H}\left(e^{j\Omega_{\mu }}\right)\right|\overset{!}{=}\bar{a}-\delta_{p,\min}\right\}\;\;\mathrm{for}\;\;\Omega_{\mu }<\Omega_{z}$$
  (21) $$\Omega_{p2}=\arg\left\{\left|\hat{H}\left(e^{j\Omega_{\mu }}\right)\right|\overset{!}{=}\bar{a}-\delta_{p,\min}\right\}\;\;\mathrm{for}\;\;\Omega_{\mu }>\Omega_{z}$$
- Stopband Edge Frequencies
  (22) $$\Omega_{s1}=\arg\left\{\left|H\left(e^{j\Omega_{\mu }}\right)\right|\overset{!}{=}\max\left(\left|\hat{H}\left(e^{j\Omega_{\mu }}\right)\right|\;\;\mathrm{for}\;\;\Omega_{\mu }<\Omega_{s1}\right)\right\}\;\;\mathrm{for}\;\;\Omega_{\mu }\ll \Omega_{p1}$$
  (23) $$\Omega_{s2}=\arg\left\{\left|H\left(e^{j\Omega_{\mu }}\right)\right|\overset{!}{=}\max\left(\left|\hat{H}\left(e^{j\Omega_{\mu }}\right)\right|\;\;\mathrm{for}\;\;\Omega_{\mu }>\Omega_{s2}\right)\right\}\;\;\mathrm{for}\;\;\Omega_{\mu }\gg \Omega_{p2}$$
- Transition Bands
  (24) $$\Delta \Omega_{1}=\Omega_{p1}-\Omega_{s1}$$
  (25) $$\Delta \Omega_{2}=\Omega_{s2}-\Omega_{p2}$$
−3 dB Angular Frequencies, Bandwidth
  (26) $$\Omega_{-3\mathrm{dB},1}=\arg\left\{\left|\hat{H}\left(e^{j\Omega_{\mu }}\right)\right|\overset{!}{=}\frac{1}{\sqrt{2}}\;\bar{a}\right\}\;\;\mathrm{for}\;\;\Omega_{s1}\leq \Omega_{\mu }\leq \Omega_{p1}$$
  (27) $$\Omega_{-3\mathrm{dB},2}=\arg\left\{\left|\hat{H}\left(e^{j\Omega_{\mu }}\right)\right|\overset{!}{=}\frac{1}{\sqrt{2}}\;\bar{a}\right\}\;\;\mathrm{for}\;\;\Omega_{p2}\leq \Omega_{\mu }\leq \Omega_{s2}$$
  (28) $$w=\Omega_{-3\mathrm{dB},2}-\Omega_{-3\mathrm{dB},1}.$$

Sensor systems with predominant resonator behavior in magnitude can be better described by resonance angular frequency ($\Omega_{\mathrm{res}}$) and −3-dB-bandwidth ($\Omega_{-3\mathrm{dB},1}$; $\Omega_{-3\mathrm{dB},2}$). These angular frequencies are related to their time-continuous counterparts $f_{\mathrm{res}}$, $f_{-3\mathrm{dB},1}$ and $f_{-3\mathrm{dB},2}$ In this case, the −3-dB-bandwidth is related to the magnitude maximum in resonance according to the condition:

(29) $$|\hat{H}\left(e^{j\Omega_{\mathrm{res}}}\right)|=1.$$

Finally, also the quality (*Q*) factor can be determined [2] corresponding to

(30) $$Q=\frac{\Omega_{\mathrm{res}}}{\Omega_{-3\mathrm{dB},2}-\Omega_{-3\mathrm{dB},1}}.$$

Other metrics are not required for an adequate resonator description. For sensor systems with predominant lowpass behavior, the provided bandpass metrics ($\bar{a}$, $\delta_{p}$, $\Omega_{p2}$, $\Omega_{s2}$, $\Delta \Omega_{2}$, $\Omega_{-3\mathrm{dB},2}$) can be modified, because only the right half of the magnitude response according to Figure 5 with $\Omega_{s1}=\Omega_{p1}=\Omega_{z}=0$ has to be considered.

#### Frequency Response Determination

**Figure 6:**
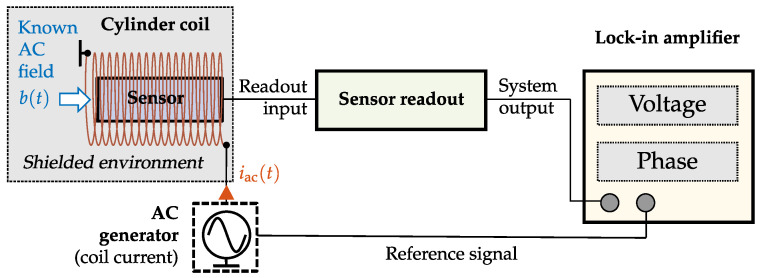
Frequency response measurement of a sensor system in a magnetically shielded environment by lock-in-amplifier, current source and cylinder coil.

Following Figure 6 the *Frequency Response* can be determined if a monofrequent signal (sinusoid) is applied in a shielded environment (permalloy cylinder) via a calibrated coil to the magnetic sensor. The current source serves as a sweep generator [30] and also as the reference signal for the lock-in-amplifier. The normalized discrete angular frequency $\Omega_{\mu }$ of the excitation signal is varied linearly in a predefined frequency range $f_{\mathrm{start}}\leq f_{\mu }\leq f_{\mathrm{end}}$. A common frequency range of biomagnetic signals extends from 0.01 Hz to 10 kHz [31]. In most biomedical applications, a supported bandwidth of approximately 1 kHz (0.01 Hz to 1 kHz) is sufficient to record fast time-dependent field variations [31]. For special applications, such as nerve activity detection and muscle spontaneous activity detection, the required bandwidth is even higher [4,32]. The excitation signal amplitude must be chosen such that the resulting magnetic flux density lies in the typical linear region (cf. Figure 2). The sensor’s sensing volume should be placed in the center of the calibrated coil. Finally, the signal at the system output is analyzed and compared to the applied AC magnetic field with regard to amplitude and phase change.

## 3. Figures of Merit for Sensor Signal Evaluation

**Figure 7:**
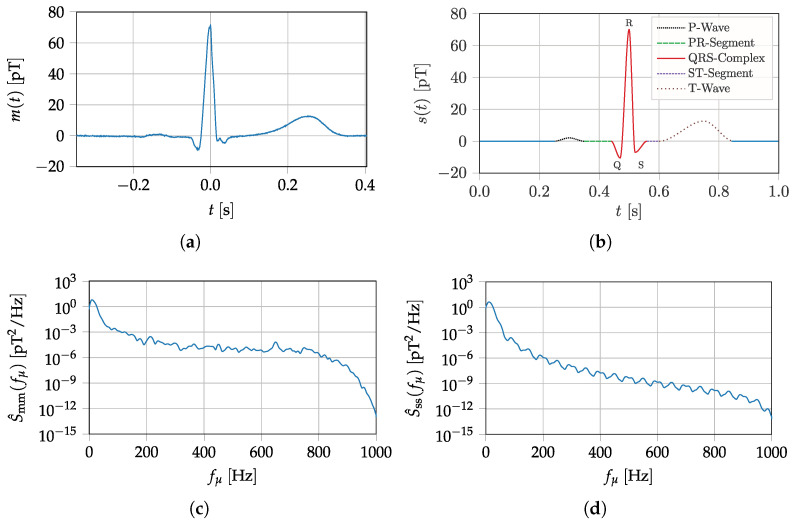
MCG prototype signal based on SQUID-MCG-Data. (**a**) SQUID Measurement—time domain. (**b**) Prototype MCG—time domain. (**c**) SQUID Measurement—power spectral density. (**d**) Prototype MCG—power spectral density.

The quantities introduced in Section 2 are targeted at comparing different sensors to each other. It is theoretically possible to evaluate the suitability of a sensor for a specific application according to these metrics. However, doing so requires experience and expertise in dealing with sensor characteristics and the application in question. In this section, we will move away from considering sensors systems on their own and start introducing metrics that can be used to evaluate them for specific applications. Therefore, figures of merit for sensor signal evaluation will be introduced, primarily influenced by the desired biomedical signal itself and the noise present in the overall system. Furthermore, an application-specific capacity is presented, which ensures a quantitative evaluation in the frequency domain. This approach is essential since diagnostic information depends mainly on signal characteristics. Therefore, a biomedical signal should remain as unaffected as possible by the sensor system; otherwise, a signal feature change will occur purely due to a technical limitation and has no pathophysiological or physiological origin. As an exemplary desired magnetic signal within the following sections, a prototype heart signal is applied. Compared to other biomedical sources like nerves or the brain, the magnetic field of the heart is by far the strongest [33]. The prototype signal is based on magnetocardiography (MCG) measurements with super conducting quantum interference device (SQUIDs) (cf. Figure 7a,c, Appendix A) recorded at the Physikalisch Technische Bundesanstalt (PTB) in Berlin. For signal generation, characteristic data points (cf. Table A1) of the SQUID-MCG recording and a cubic Hermite spline are applied. Using a sampling frequency of $f_{s}=2000\;\mathrm{Hz}$ results in the signal s(t) (cf. Figure 7b,d, which is used in the following experiments. At low frequencies, the PSDs of the prototype and SQUID signals are very similar. The deviations at higher frequencies might be explained by the absence of additive noise in the prototype signal. For the estimation of the PSD, Welch’s method is used in this section with a signal length of 5 s, a Hanning window of 256 samples width, an overlap of 50 percent, and an FFT length of 4096. In the next section, the system noise is introduced, which is fundamental for all upcoming metrics.

### 3.1. System Noise

The desired biomagnetic signal is usually superimposed by undesired noise. These stochastic signal components can be characterized in the frequency domain with the frequency-dependent PSD. As a consequence, PSD measurements are performed after the required post-processing steps, e.g., *filtering*, *demodulation*, and *A/D conversion*, in order to evaluate the entire noise characteristics (cf. Figure 1). A noise-free system could theoretically acquire arbitrarily small measurement signals and optimally adapt them to the dynamic range of digitization. The detection limit of sensor systems is constrained by noise processes present at the system output, whereby a distinction of the noise sources is not considered in this analysis. This aggregated noise describes unwanted signals and processes of all components within the signal chain, which results in decisive limitations [15]. The PSD for a stationary random process can be determined from the Fourier Transform of the autocorrelation function (ACF) by applying the Wiener–Khintchine theorem [18]. In practice, for PSD estimation ${\hat{S}}_{\mathrm{xx}}\left(\Omega_{\mu }\right)$ of a digitized sequence x(n), the well-known Welch’s algorithm [34] is mainly used, where $\Omega_{\mu }$ describes the normalized frequency bins (Furthermore, using the sampling frequency $f_{s}$ and the relation $f_{\mu }=\Omega_{\mu }\;\cdot \;f_{s}/\left(2\pi \right)$, the estimated power spectral density can also be related to the discrete frequency bins $f_{\mu }$). Welch’s algorithm guarantees a reduction of the variance in the frequency domain based on multiple windowed and squared Discrete Fourier Transform (DFT, *periodograms*) averages. In order to ensure traceability of the results, acquisition time and the total amount of averages should be provided. The resulting power density spectrum estimated from the noise is called noise power spectral density and allows the additive superposition of uncorrelated noise sources. Typically for sensor system specification, the amplitude spectral density (ASD) is provided instead, which is the square root of the power density spectrum $\sqrt{{\hat{S}}_{\mathrm{xx}}\left(\Omega_{\mu }\right)}$. It represents the RMS value as a physical unit of the measurand with respect to a frequency bandwidth of 1 Hz [24]. Moreover, the density spectra (PSD/ASD) are related to the common power spectra and amplitude spectra (PS/AS) via the equivalent noise bandwidth (ENBW) [20]. At the output of a sensor system, voltages are directly acquired so that the noise power density spectrum in V^2^/Hz can be represented as amplitude spectral density,

(31) $$\sqrt{{\hat{S}}_{\mathrm{uu}}\left(\Omega_{\mu }\right)},$$

in the unit V/$\sqrt{\mathrm{Hz}}$. On the other hand, magnetic noise,

(32) $$\sqrt{{\hat{S}}_{\mathrm{bb}}\left(\Omega_{\mu }\right)},$$

is given in the unit T/$\sqrt{\mathrm{Hz}}$ [15]. Finally, to guarantee a unit conversion from the electric output quantity to the magnetic input quantity, a description of the overall system *sensitivity*$\epsilon_{\mathrm{sys}}$ is necessary in the unit V/T [19]. The frequency-dependent sensitivity $\epsilon_{\mathrm{sys}}\left(\Omega_{\mu }\right)$ is the ratio of the output voltage to the change of a known predefined magnetic flux density ($B_{\mathrm{ext}}\neq 0$) so that the following equation holds:

(33) $$\epsilon_{\mathrm{sys}}\left(\Omega_{\mu }\right)=\frac{U_{\mathrm{out}}\left(\Omega_{\mu }\right)}{B_{\mathrm{ext}}\left(\Omega_{\mu }\right)},$$

whereby $U_{\mathrm{out}}\left(\Omega_{\mu }\right)$ denotes the RMS sensor output voltage and $B_{\mathrm{ext}}\left(\Omega_{\mu }\right)$ the RMS magnetic flux density as input quantity at a particular normalized angular frequency $\Omega_{\mu }$. It should be mentioned that only a sensitivity determination is performed to get the also required physical unit conversation factor, while the frequency response (cf. Equation (15)) is usually dimensionless. After all, the magnetic field noise can be determined in the unit T/$\sqrt{\mathrm{Hz}}$. Therefore, the noise voltage spectral density is divided by the frequency-depended sensor sensitivity according to

(34) $$\sqrt{{\hat{S}}_{\mathrm{bb}}\left(\Omega_{\mu }\right)}=\frac{\sqrt{{\hat{S}}_{\mathrm{uu}}\left(\Omega_{\mu }\right)}}{\epsilon_{\mathrm{sys}}\left(\Omega_{\mu }\right)}.$$

Thus for the achievement of an overall low magnetic field noise density, high sensitivity and low noise are required. The specification of the noise as a parameter must always be related to the bandwidth *w*. The *effective magnetic noise amplitude* $b_{n}^{\mathrm{rms}}\left(\Omega_{-3\mathrm{dB},1},\Omega_{-3\mathrm{dB},2}\right)$, which is available within a given bandwidth (−3 dB or −6 dB sensor bandwidth are commonly used, cf. Equations (26) and (27)), can be determined from the estimated frequency-dependent power spectral density ${\hat{S}}_{\mathrm{bb}}\left(\Omega_{\mu }\right)$ by:

(35) $$\begin{array}{c} \begin{array}{cc} b_{n}^{\mathrm{rms}}\left(\Omega_{-3\mathrm{dB},1},\Omega_{-3\mathrm{dB},2}\right) & =2\;\cdot \;\sqrt{\lim_{\Delta \Omega_{\mu }\to 0}\sum_{\Omega_{\mu }=\Omega_{-3\mathrm{dB},1}}^{\Omega_{-3\mathrm{dB},2}}{\hat{S}}_{\mathrm{bb}}\left(\Omega_{\mu }\right)\;\cdot \;\Delta \Omega_{\mu }}\\ \; & \forall \;\;\;0\leq \Omega_{-3\mathrm{dB},1}\leq \Omega_{\mu }\leq \Omega_{-3\mathrm{dB},2}. \end{array} \end{array}$$

**Figure 8:**
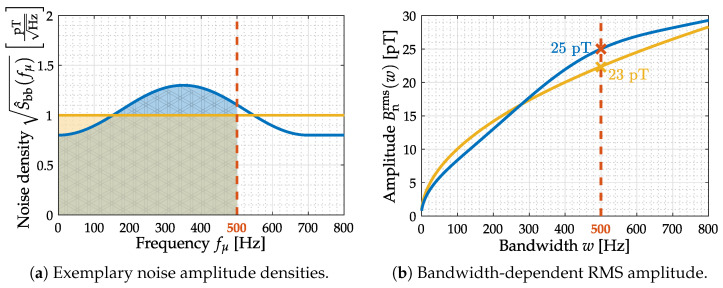
Summation of noise amplitude densities. Two exemplary noise amplitude densities (constant and arbitrary shape) are provided (**a**). Summation is performed from DC up to an increasing upper cutoff frequency to obtain the corresponding RMS value (**b**) for both densities. Assuming a sensor −3 dB cutoff frequency of 500 Hz, the colored areas under curve (**a**) yield RMS amplitudes of 25 pT and 23 pT, which will vary if a different upper frequency limit is applied (**b**).

The lower normalized angular cutoff-frequency $\Omega_{-3\mathrm{dB},1}$ and the upper normalized angular cutoff-frequency $\Omega_{-3\mathrm{dB},2}$ are quite crucial for effective noise amplitude determination. Therefore, a bandwidth reduction usually results in a decrease in noise amplitude. Figure 8 illustrates the ambiguity of this metric without a given bandwidth specification.

Consequentially, the considered frequency range/bandwidth is another characteristic value provided for effective noise amplitude considerations. Please note that the noise consideration within 3-dB-bandwidth is meant as a general sensor system performance metric. Any practical (biomedical) application might produce varying noise characteristics due to its respective bandwidth requirements and application-specific prefilters.

#### Measurement

The overall system noise n(t) of a sensor system can be determined with a minor change of the experimental setup shown in Figure 6. For this purpose, the external magnetic excitation, including the coil, is no longer required and should be removed entirely from the experimental setup to avoid unnecessary additional noise components. Finally, the sensor is operated in an almost zero field environment b(t)≈0, for example, in a permalloy cylinder, and the system output voltage is continuously analyzed.

### 3.2. Signal-to-Noise Ratio

A quantity commonly used to describe signal quality is the SNR. The SNR quantitatively describes the differences in power between signal and noise by the quotient: (36)
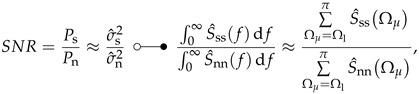

where $P_{s}$ is the average power of the signal and $P_{n}$ is the average power of the noise. The SNR could also be estimated by the ratio between estimated signal variance ${\hat{\sigma }}_{s}^{2}$ and estimated noise variance ${\hat{\sigma }}_{n}^{2}$ of the time domain signals. Another approximation could be made in the spectral domain by using the application-specific PSD of the signal ${\hat{S}}_{\mathrm{ss}}\left(\Omega_{\mu }\right)$ and the noise PSD of the sensor ${\hat{S}}_{\mathrm{nn}}\left(\Omega_{\mu }\right)$ (cf. Equation (34)).

Since it is not possible to measure the pure signal component in the absence of noise, it can be more practical to calculate the signal-plus-noise to noise ratio [35] (*SNNR*) instead: (37)
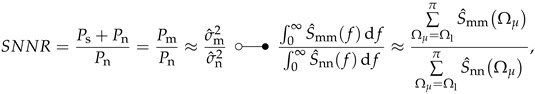

where $P_{m}$ is the average power of the measured signal (superimposed with the noise), $\sigma_{m}^{2}$ is the estimated variance of the measured signal and ${\hat{S}}_{\mathrm{mm}}\left(\Omega_{\mu }\right)$ is its estimated power spectral density. The *SNNR* contains the same information as the *SNR*. In order to convert one into the other, the following relationship can be used:

(38) $$SNNR=\underset{SNR}{\underset{︸}{\frac{P_{s}}{P_{n}}}}+\frac{P_{n}}{P_{n}}=SNR+1.$$

**Figure 9:**
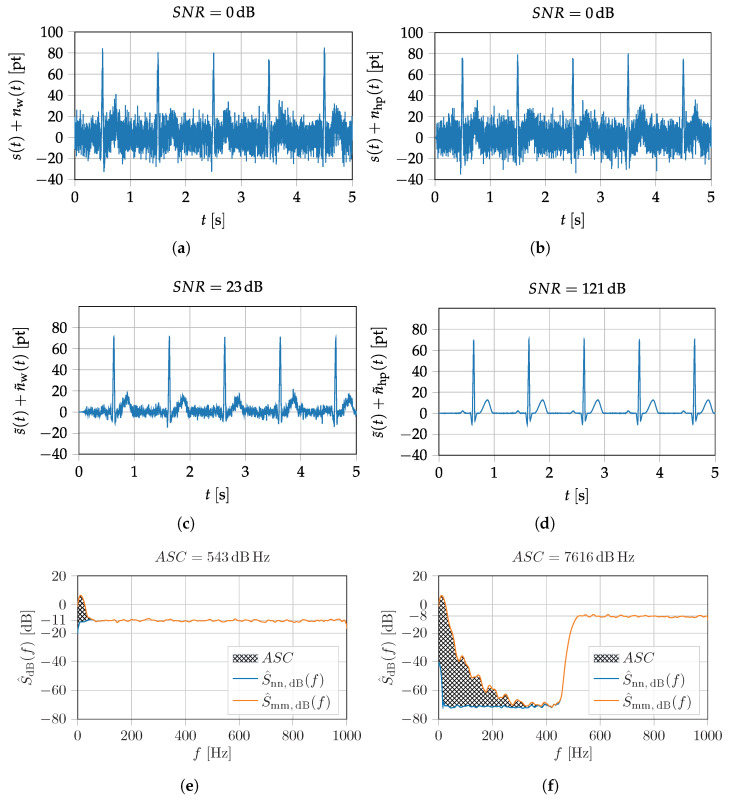
MCG-Prototype signals (cf. Figure 7b) superimposed with white noise (left column) and high-pass filtered noise (right column). The second row shows the low-pass filtered sum of the signal and noise, while the third row shows application specific capacity and the power spectral densities of the signal, noise and weighted noise (cf. Equation (39)). (**a**) MCG Signal plus white noise—time signal. (**b**) MCG Signal plus HP noise—time signal. (**c**) MCG Signal plus white noise—time signal, filtered. (**d**) MCG Signal plus HP noise—time signal, filtered. (**e**) MCG Signal and white noise—PSD. (**f**) MCG Signal and HP noise—PSD.

To calculate the power from the PSD it is theoretically necessary to integrate from *f* = 0 Hz to f→∞. Since this calculation takes place digitally in practice, some approximations and restrictions have to be made. First, the integration over the frequency becomes a numerical integration over the support points $\Omega_{\mu }$ and the upper integration limit is confined to $\Omega_{u}=\pi$, due to the sampling theorem. In practice, the lower integration limit can be confined by metrological constraints to a value of $\Omega_{l}$. In the following simulations, Romberg’s method is used for the numeric integration and $\Omega_{l}=0$. The main problem of the SNR metric is explainable with Figure 9a,b. Both signals look qualitatively the same, but in one case the introduced prototype MCG-signal (cf. Figure 7b) is superimposed with white noise $n_{w}\left(t\right)$ and in the other case with high-pass (HP) filtered noise $n_{\mathrm{hp}}\left(t\right)$. A frequency-independent performance metric like SNR does not sufficiently consider the ability of a filter to improve the signal quality by separating the desired and undesired signal components.

For the electromagnetic field of the human heart, it is known that the signal contains no significant power above frequencies of 100 Hz (cf. Figure 9c,d) [36,37]. Therefore, applying a suitable band limitation by a low-pass filter (FIR filter using the Remez exchange algorithm [38] with N=516; $\mathrm{bands}=\left[0,100,110,1000\right]\;\mathrm{Hz}$; $\mathrm{normalized}\;\mathrm{gain}=\left[1,1,1\times 10^{-4},1\times 10^{-4}\right].$), reveals that the high frequency noise can be easily suppressed, while the white noise can only be partially suppressed. In this particular example this results in two different superimposed signals, which had the same SNR (0 dB) at the beginning, but look very different after filtering (cf. Figure 9c,d). Calculating the SNR after applying the low-pass filter yields an SNR of 23 dB in the case of white noise and 121 dB in the case of high frequency noise.

Furthermore, the applied sampling frequency also influences the SNR result, because the entire frequency interval between 0 and $f_{s}/2$ is considered by default (cf. Equation (36)). The desired signal only has relevant components within a specific bandwidth, that are necessary to preserve the signal characteristics for diagnostic proposes. Increasing the sampling frequency will worsen the SNR, while in practice, a filter can be applied to limit the signal to the appropriate bandwidth. For a consistent system evaluation, the influence of the signal bandwidth and the spectral characteristics must be considered. A figure of merit used to describe potential signal quality after processing needs to either explicitly consider post-processing steps (i.e., applying the same band limitation to signal and noise) or take the frequency dependence of the PSDs into account. Since the required processing steps depend highly on the system output and the specific biomedical application, a metric that focuses on the individual power spectral densities and their predefined frequency limits is preferable.

### 3.3. Application Specific Capacity

To take the frequency-dependent power into account, a suitable type of operation has to be applied on the PSDs before integration (cf. Equation (36)). The strength at which we consider the power of the noise at a certain frequency should be dependent on the power of the signal ${\hat{S}}_{\mathrm{ss}}\left(\Omega_{\mu }\right)$ at that particular normalized frequency $\Omega_{\mu }$. If ${\hat{S}}_{\mathrm{ss}}\left(\Omega_{\mu }\right)$ is low at a certain frequency, that frequency can be filtered out of the measurement without distorting the signal—resulting in a better signal quality. Therefore, high noise power at frequencies where the signal power is low should not negatively influence the quantity. High noise power at frequencies where the signal power is also significant, on the other hand, should reduce the quantity. The spectral contribution at those frequencies can not be removed from the measurement without disturbing the desired signal. This required constraint can be achieved by integrating over the logarithm of the ratio between the power spectral densities of the measured and the desired signal. Doing so results in an equation that is identical (besides a different basis of the logarithm and additional scaling) to that of the channel capacity *C* [39], which is why we introduce the term *Application Specific Capacity* and the symbol ASC for this quantity:

(39) $$\begin{array}{cc} ASC & =\int_{0}^{\infty }10\;\cdot \;\log_{10}\left(\frac{{\hat{S}}_{\mathrm{ss}}\left(f\right)+{\hat{S}}_{\mathrm{nn}}\left(f\right)}{{\hat{S}}_{\mathrm{nn}}\left(f\right)}\right)\;df\;\cdot \;\mathrm{dB}\\ \; & \approx \frac{1}{\Omega_{u}-\Omega_{l}}\;\sum_{\Omega_{\mu }=\Omega_{l}}^{\Omega_{u}}10\;\cdot \;\log_{10}\left(\frac{{\hat{S}}_{\mathrm{ss}}\left(\Omega_{\mu }\right)+{\hat{S}}_{\mathrm{nn}}\left(\Omega_{\mu }\right)}{{\hat{S}}_{\mathrm{nn}}\left(\Omega_{\mu }\right)}\right)\;\mathrm{dB}\\ \; & \approx \frac{1}{\Omega_{u}-\Omega_{l}}\;\sum_{\Omega_{\mu }=\Omega_{l}}^{\Omega_{u}}10\;\cdot \;\log_{10}\left(\frac{{\hat{S}}_{\mathrm{mm}}\left(\Omega_{\mu }\right)}{{\hat{S}}_{\mathrm{nn}}\left(\Omega_{\mu }\right)}\right)\;\mathrm{dB}, \end{array}$$

where ${\hat{S}}_{\mathrm{mm}}\left(\Omega_{\mu }\right)$ is the PSD of the measured signal, $\Omega_{u}$ is the upper and $\Omega_{l}$ the lower normalized frequency limit of the numeric integral with $\Omega_{-3\mathrm{dB}}<\Omega_{u}\leq \pi$. For the calculation of the ASC, the same considerations to PSD estimation and integration as mentioned in Section 3.2 apply. In the following simulations $\Omega_{l}=0$ and $\Omega_{u}=\pi$ is used. To understand how this equation satisfies the abovementioned constraints, consider the following: In regions where ${\hat{S}}_{\mathrm{nn}}\left(\Omega_{\mu }\right)$ is greater than ${\hat{S}}_{\mathrm{ss}}\left(\Omega_{\mu }\right)$, the ratio between ${\hat{S}}_{\mathrm{mm}}\left(\Omega_{\mu }\right)$ and ${\hat{S}}_{\mathrm{nn}}\left(\Omega_{\mu }\right)$ will be close to one. Consequently, the logarithm of the ratio will be close to zero. These regions, therefore, do not contribute significantly to the ASC. If the signal power ${\hat{S}}_{\mathrm{ss}}\left(\Omega_{\mu }\right)$ is high while the noise power ${\hat{S}}_{\mathrm{nn}}\left(\Omega_{\mu }\right)$ is low, the dB power difference will be big, resulting in a significant contribution to the ASC.

Considering the ASC beyond the necessary bandwidth (determined by the bandwidth of the desired signal) will not noteably affect the ASC. This is a desired behavior since a filter can always be applied to the measured signal afterwards to reduce its bandwidth. In practical terms, this means that considering the PSD of the noise over a wider range of frequencies will not significantly change the ASC. Compared to the SNR this eliminates one potential cause for inconsistencies between different measurements.

Taking a look at the ASC values for the previous example (cf. Figure 9), it can be seen that the ASC exhibits the desired behavior. For the case of white noise the ASC equals 543 dB Hz and for the case of high-frequency noise, it is 7616 dB Hz. The SNR of the input signals is 0 dB in both cases. Consequently, after processing the signal superimposed with the high-frequency noise, it could have a better quality than the signal superimposed with the white noise (provided that the applied processing is sensible). This is in accordance with the results of the previous section (cf. Figure 9c,d).

## 4. Exemplary Evaluation of Magnetoelectric Sensor Systems

**Figure 10:**
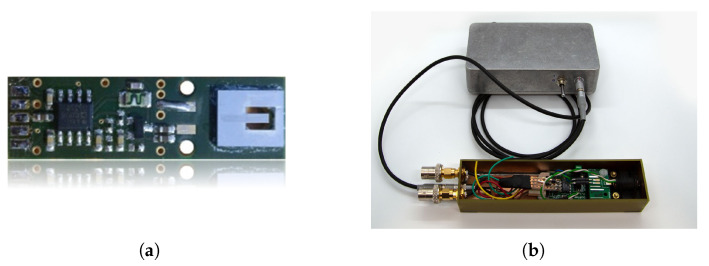
Sensors systems used in this study: In (**a**) an exchange bias magnetoelectric sensor is shown with integrated readout electronics. In (**b**) an electrically modulated ME sensor is presented with integrated preamplifier and external shielded battery supply (gray box; ±9 V). (**a**) Exchange bias magnetoelectric sensor (cantilever) with integrated readout. (**b**) Electrically modulated ME sensor with integrated preamplifier and external battery.

In this section, two different ME sensor systems will be assessed by applying the functional characteristics proposed in Section 2 and Section 3. Both sensor concepts are investigated at Kiel University. The *exchange bias magnetoelectric sensor* is used to demonstrate a typical resonant ME sensor system. This sensor type is especially applicable for detecting narrowband signals, for example, coil signals utilized in novel ME localization [12] and ME movement detection applications [13]. In contrast to this, the *electrically modulated ME sensor* is potentially better suited for broadband biomedical signals due to a much higher bandwidth. Both sensors are shown in Figure 10 and their concepts will be separately introduced and evaluated in the following subsections. In addition, the SNR and the ASC, presented in Section 3.2 and Section 3.3, are used as a figure of merit concerning a possible sensor usage for MCG. Therefore, the definitions are applied by using the noise measurements and the generated prototype MCG signal (cf. Figure 7b) with its spectral distribution. Both sensor systems will be compared and finally discussed in a results overview.

The measurements for evaluation have been performed in a magnetically, electrically, and acoustically shielded environment comprising a multilayer mu-metal cylinder (Model ZG1 from Aaronia), further details are given in [11,40]. The noise measurements have been accomplished with the Dynamic Signal Analyzer SR785 from Stanford Research Systems [41]. All other measurements, where a magnetic signal is required, have been performed with a long solenoid driven by the low noise current source Keithley 6221 [30]. The coil was used to generate a mono-frequent signal with a magnetic field amplitude of $b_{\mathrm{in}}^{\mathrm{rms}}=1$ µT (desired signal). The amplitude and phase responses, as well as the linearity curve of the sensors, have been measured with the lock-in amplifier SR830 from Stanford Research Systems [42]. For determining the linearity curve, the amplitude of the magnetic flux density within the solenoid was varied in the range from 0.1 pT to 100 μT. Consequently, the coil excitation signal has been used as the reference signal for the lock-in amplifier and the acquisition time was set to 100 ms.

In addition, it is essential to ensure a dedicated magnetic state of the ME sensor before the sensor system evaluation starts, especially considering hysteresis effects of the magnetostrictive layer. Magnetic saturation of the magnetostrictive layer can be achieved using a high constant field within the coil. Therefore, a DC current source (BOP 20-10ML from KEPCO) is used. The direction of magnetic saturation is sensor dependent and was be chosen such that the best sensor performance in terms of sensitivity and noise is reached. Finally, this dedicated magnetic state served as the starting point for the ME sensor system evaluation.

### 4.1. Exchange Bias Magnetoelectric Sensor

**Figure 11:**
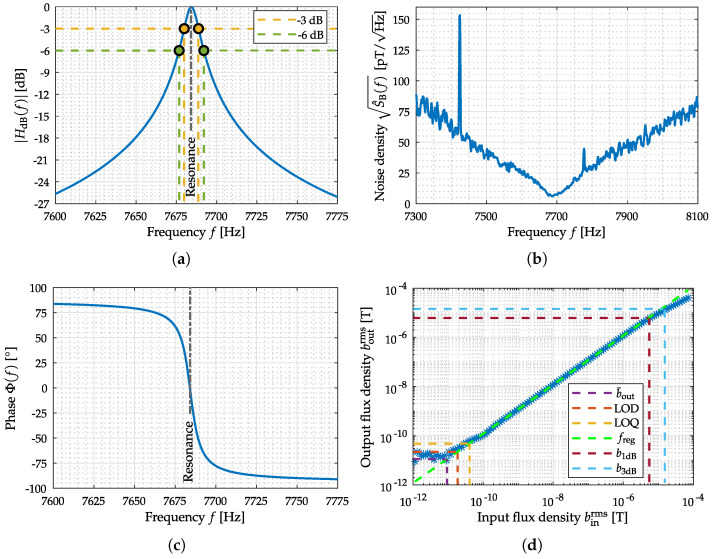
Measurements for the evaluation of the exchange bias magnetoelectric sensor system. In (**a**) the amplitude response and in (**c**) the phase response of the sensor system near the mechanical resonance are depicted. The noise measurement equalized with amplitude response is shown in (**b**). The Input–Output–Amplitude–Relation of the sensor, with an external magnetic field at *f* = $f_{\mathrm{res}}$, is depicted in (**d**).

ME thin film composite sensors are composed of mechanically coupled magnetostrictive and piezoelectric layers and utilize the mechanical resonance of a cantilever structure [11]. Hence, a resonator behavior (bandpass characteristic) is present when operating the sensor in direct detection, that is, without any modulation technique. Besides reading out the sensor directly in its mechanical resonance, various readout schemes can be applied to the sensor for measuring low-frequency signals. Recently investigated readout schemes for ME sensors are e.g., the ΔE-effect [43,44,45] or magnetic frequency conversion [46,47]. In this contribution, an exchange bias ME sensor in a so-called direct detection mode has been used as shown in Figure 10a. The cantilever sensing element has a size of 3 mm × 1 mm × 0.1 mm. The sensor is connected to a low-noise JFET (junction-gate-field-effect transistor) charge amplifier [48]. Due to the exchange biasing of the sensor, there is no need for an additional coil generating a magnetic bias field [49]. Further details about the sensor and the fabrication process can be found in [50]. The sensor was operated in direct detection and the output signal of the sensor system, including the charge amplifier, was taken into account. For comparability with the other ME sensor type (shielded printed-circuit-board (PCB) housing, cf. Figure 10b), this sensor is also operated with additional shielding. Therefore, the sensor has been provided with an extra electromagnetic compatible (EMC) braided cable and has been connected to the measurement ground. A sensor operation from negative saturation showed the best sensor performance at the resonance frequency in terms of sensitivity and noise. Therefore, the ME sensor was saturated before the ME system evaluation. Three representative measurements have been performed for the evaluation of this particular sensor system. The *amplitude* and *phase response*, the *noise spectrum*, and the *Input–Output–Amplitude–Relation* are shown in Figure 11. For determining the noise amplitude spectral density a frequency range of 800 Hz was observed around $f_{\mathrm{res}}$ with an FFT size (single-sided) of 800 points, resulting in a frequency resolution of 1 Hz and a total acquisition time of 1 s (60 RMS averages). In addition, especially for determining SNR and ASC, a noise amplitude spectral density from 4 to 800 Hz (the same FFT size) has been acquired with an identical acquisition time of 1 s (60 RMS averages) to cover the required MCG-Bandwidth (cf. Figure 7d). The Input–Output–Amplitude–Relation measurements have been performed in resonance of the sensor at $f_{r}=7684$ Hz. As stated with the help of Figure 3 for a reproducible LOD determination an exact specification of the measurement routine is required. Here, a dedicated RC-lowpass filter with a slope of 24 dB/oct and a time constant of 100 ms have been chosen at the lock-in amplifier [42].

The expected resonator behavior of the ME sensor is visible in the amplitude spectrum in Figure 11a. The noise spectrum of the sensor is dominated by thermal-mechanical noise [51] as shown in Figure 11b. Looking at the Input–Output–Amplitude–Relation in Figure 11d, the quantities ${\bar{b}}_{\mathrm{out}}=11$ pT, LOD=22pT and LOQ=42pT can be determined. Furthermore, the linear region can be described by using a linear function approximation $f_{\mathrm{reg}}$. Based on the magnetic hysteresis, nonlinearities occur bevor reaching the operating voltage (±12V), so it is helpful to determine the compression points from the Input–Output–Amplitude–Relation. The *1-dB-compression-point* ($b_{1\mathrm{dB}}$) is at $b_{\mathrm{in}}^{\mathrm{rms}}\approx 5.8$ µT and the *3-dB-compression-point* ($b_{3\mathrm{dB}}$) is at $b_{\mathrm{in}}^{\mathrm{rms}}\approx 17.6$ µT. Based on the noise spectral measurement covering the bandwidth from 4 to 800 Hz and the applied prototype MCG signal, an SNR of −90 dB and an ASC of 9.8 $\times 10^{-7}$ dB Hz could be determined quantitatively.

### 4.2. Electrically Modulated ME Sensor

Resonant magnetoelectric (ME) sensors combined with modulation techniques can be used to achieve high bandwidth at low frequencies. Consequently, it is favorable to use electric instead of magnetic modulation. Magnetic modulation has demonstrated its general high potential but suffers from high power consumption and possible crosstalk between sensors. For alternative ME sensor concept realization, the piezoelectric phase of thin film magnetoelectric composites is actively excited by an alternating voltage, thus exploiting the converse ME effect [10], remedying shortcomings of the direct ME effect. High frequency mechanical resonances between 500 kHz and 540 kHz are typically used for sensor operation. These resonances show high mechanical quality factors (Q ≈ 1000) [10], which results in better SNRs at those frequencies. The resulting mechanical oscillation, being rigidly coupled into the magnetostrictive material phase leads to a voltage induced in a pickup coil surrounding the sensor composite. This converse ME voltage response with respect to small external fields shows high sensitivities in the order of kV/T. No external magnetic driving field is required, as is the case for the exchange bias ME sensor using magnetic frequency conversion techniques.

The ME sensor system (shielded housing) is shown in Figure 10b with the output preamplified by a low-noise operational amplifier (LT1128) in unity gain configuration to decouple the resonant circuit from the readout. This operational amplifier is connected to the additional shielding box that contains a ±9 V battery powered voltage supply and has been connected to the measurement ground. Further details about this particular ME sensor type and the fabrication process can be found in [9].

The electrically modulated ME sensor (cf. Figure 10b) is piezoelectrically excited at 514.249 kHz (2nd mechanical U-mode of oscillation). Therefore, a sinusoidal voltage with an amplitude of 500 mV has been used. Sensor excitation and the required synchronous demodulation of the coil signal are performed using a high frequency lock-in amplifier (HF2LI from Zurich Instruments). A 4th-order RC-lowpass filter (24 dB/oct or 80 dB/dec) with a cutoff frequency of 30 kHz has been chosen as the demodulation filter within the lock-in amplifier. The demodulated analog coil signal (lock-in amplifier output) is used with an additional output gain of one hundred for signal acquisition to optimally use the internal A/D converter dynamics. The results have been corrected for the applied amplification factor to show the correct (unamplified) values. The amplitude and phase response of the sensor have been measured between 1 Hz and 100 Hz with a resolution of 1 Hz and between 100 Hz and 30 kHz with a resolution of 100 Hz. The noise amplitude spectral density with a frequency range from 16 Hz to 12.656 kHz was determined with a FFT size of 800 points, resulting in a frequency resolution of 16 Hz and a total acquisition time of 1 min (960 RMS averages). For determining the SNR and ASC the noise amplitude spectral densities over the important frequency range from 4 to 800 Hz (same FFT size) has been acquired with an identical acquisition time of 1 min (60 RMS averages). The acquisition of the Input–Output–Amplitude–Relation has been performed changing the flux density of the external magnetic field at a frequency of 10 Hz. The measurements performed for evaluation are the three measurements shown in Figure 11, the *amplitude* and *phase response*, the *noise spectrum*, and the *Input–Output–Amplitude–Relation*.

**Figure 12:**
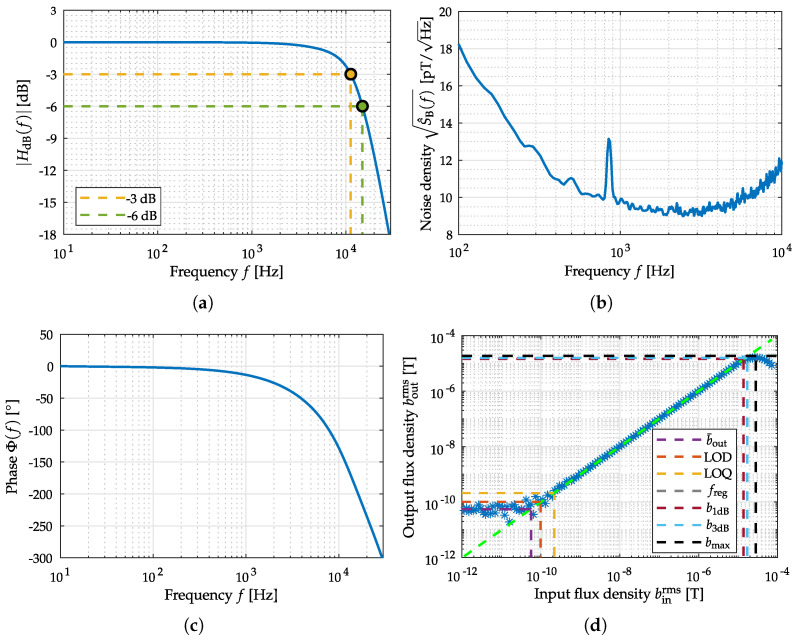
Measurements for the evaluation of the electrically modulated ME sensor. In (**a**) the amplitude response and in (**c**) the phase response of the sensor system are depicted. The noise measurement equalized with amplitude response is shown in (**b**). The Input–Output–Amplitude–Relation of the sensor, with an external magnetic field at *f* = 10 Hz, is depicted in (**d**).

By analyzing the amplitude response in Figure 12a, the lowpass-characteristic of the sensor with a larger supported signal bandwidth is visible. The phase response in Figure 12b is linear (even if being depicted with a logarithm scaled *x*-axis). Figure 11b shows the noise amplitude density spectrum of the sensor, where the excessive magnetization reorientation initiated by stress anisotropy and the emergence of eddy currents are the main known noise sources [10]. By using the Input–Output–Amplitude–Relation (*f* = 10 Hz) the defined metrics can be determined: ${\bar{b}}_{\mathrm{out}}$ is 55 pT, LOD is 102 pT and LOQ is 210 pT. Saturation from external magnetic flux density after $b_{\mathrm{in}}^{\mathrm{rms}}\approx 26.70$ µT generate a decay in the output flux density. Therefore, the *1-dB-compression-point* ($b_{1\mathrm{dB}}$) and *3-dB-compression-point* ($b_{3\mathrm{dB}}$) could be determined to $b_{\mathrm{in}}^{\mathrm{rms}}\approx 18$ µT and $b_{\mathrm{in}}^{\mathrm{rms}}\approx 23$ µT. Based on the noise spectral measurement covering the bandwidth from 4 to 800 Hz and the applied prototype MCG signal, an SNR of −11 dB and an ASC of 23 dB Hz could be determined.

### 4.3. Overview of the ME Evaluation Results

A comparison of the sensor systems evaluated within this contribution is provided in the following. Table 2 shows an overview of the essential sensor system metrics and also the application-specific values concerning an ME sensor system usage in cardiovascular medicine. By considering SNR and ASC, it can be determined if the sensor system can reliably detect a magnetic heart signal. As described in Section 4.3, the parameters used for estimating the PSDs and integrating them are of significant importance for the comparability of the results. Since the PSDs of the sensors are determined by measurement and those of the signals by simulation, the parameters of the simulation must be adapted to those of the measurement. The estimation of the PSD of the prototype signal is therefore done by Welch’s method using a signal length of 5 s, a Flattop window of 1600 samples width, an overlap of 50 percent, and an FFT length of 1600. For the numerical integration Simpson’s rule with the limits $f_{l}=4\;\mathrm{Hz}$ and $f_{u}=f_{s}/2=800\;\mathrm{Hz}$ is used. The upper cutoff frequency does not have a significant effect on the ASC here, since the prototype signal has no significant components above this frequency. The lower cutoff frequency of 4 Hz, limited by the measurement setup, on the other hand, affects both SNR and ASC. Since this cutoff frequency was used identically for the measurements of both sensors, the results remain comparable here. In a comparison with other measurements or simulations; however, care would have to be taken to maintain the same integration limits.

Beginning with the exchange bias ME sensor, it is evident that the frequency range where the sensor is operating is too high and thus not compatible with the bandwidth of a magnetically detected heart signal. A frequency range from 0.01 to 100 Hz [31] is typically required for a signal-true MCG recording. This bandwidth specification could also be confirmed with an MCG performed with SQUIDs. In Figure 7, the time signal (a) and the resulting power spectral density (c) of a single MCG heartbeat recorded by a SQUID have been presented. Accordingly, this ME sensor system is not suitable for measuring heart signals when operating in direct detection. This can also be quantitatively confirmed with the given SNR and ASC. Nevertheless, this sensor type enables detecting narrowband signals with a center frequency of 7684 Hz, for example, modulated coil signals applied for ME localization and movement detection. Furthermore, using a modulation technique (e.g., ΔE-effect, magnetic frequency conversion), the sensors can measure low-frequency magnetic fields. In [33] magnetoelectric sensors have been evaluated concerning cardiologic applications. For example, the R-wave could be detected, averaging the time signal over 743 periods by using magnetic frequency conversion for ME sensor readout. Different signal enhancement stages for improving the quality of an MCG using uncooled magnetometers are additionally applicable as a post-processing step [52], but they have not to be considered primary for sensor evaluation.

Evaluating the electrically modulated ME sensor, it is evident that the bandwidth of the sensor is quite large in direct comparison to the ME sensor operating in direct detection. Therefore, more noise is picked up by the sensor, which results in an RMS noise amplitude of approximately 12 nT within bandwidth (1 Hz to $f_{-3\mathrm{dB}}$). For the Input–Output–Relation, in general, only a mono-frequency consideration is performed at 10 Hz, and the extracted metrics were in good agreement with already published ones. When considering the available bandwidth and the LOD, the electrically modulated ME sensor is close to measuring a magnetic heart signal under ideal conditions [9]. This can be quantitatively confirmed by considering SNR (−11 dB) and ASC (23 dB Hz). The dominant noise source of this kind of sensor is the intense magnetization activity, practically limiting the LOD. Using sophisticated magnetic layer systems such as exchange bias have already shown to effectively lower the magnetically dominated noise [53], while maintaining the sensor performance. Finally, this also enables the possibility to bring magnetoelectric thin film sensors towards a cardiovascular application.

## 5. Discussion

**Figure 13:**
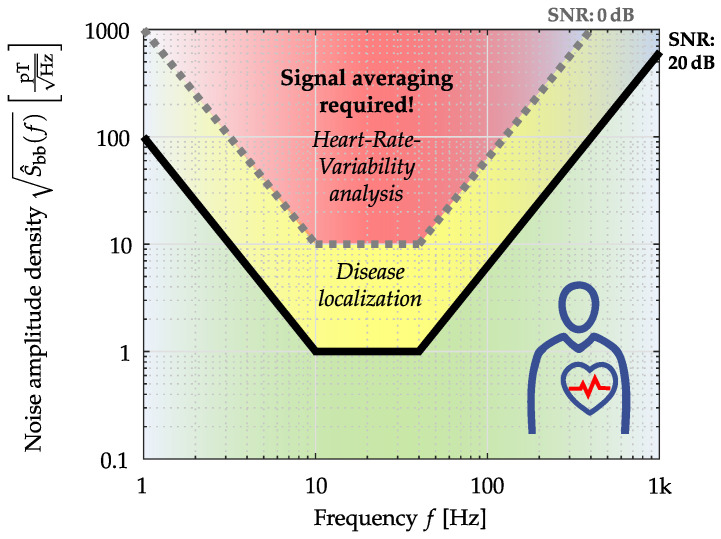
Application-specific noise requirements for MCG (Heart-Rate-Variability analyses by detection of the R-Peak; Disease localization by solving the inverse problem) specified by the amplitude density. Sensor positioned directly over the chest [55].

Based on the proposed application-oriented comparison, two different ME sensor systems have been evaluated and compared here. First, the ME sensor systems were rated with common metrics, and additionally, a signal evaluation was performed for a cardiovascular application. While targeted at ME sensors here, the same methodologies are applicable to any magnetometer. By exemplary, applying the introduced evaluation methods, it can be concluded that especially the *electrically modulated ME sensor system* has the potential to measure a magnetic heart signal, at least by applying advanced averaging techniques [52]. The other presented ME sensor type is better suited for applications where only a small frequency range is necessary, such as active magnetoelectric motion sensing [13] or ME sensor localization [12]. Nevertheless, cardiovascular medicine requires an unaveraged MCG morphology (QRS complex, P-wave, and T-wave) in the time domain, especially as cardiac arrhythmias manifest from beat to beat [7,54]. For this reason, ME sensor systems (sensor and readout electronics) are currently being further developed interdisciplinarily to ensure their use in cardiology [9]. In Figure 13, an application-specific noise requirement for MCG is presented.

**Figure 14:**
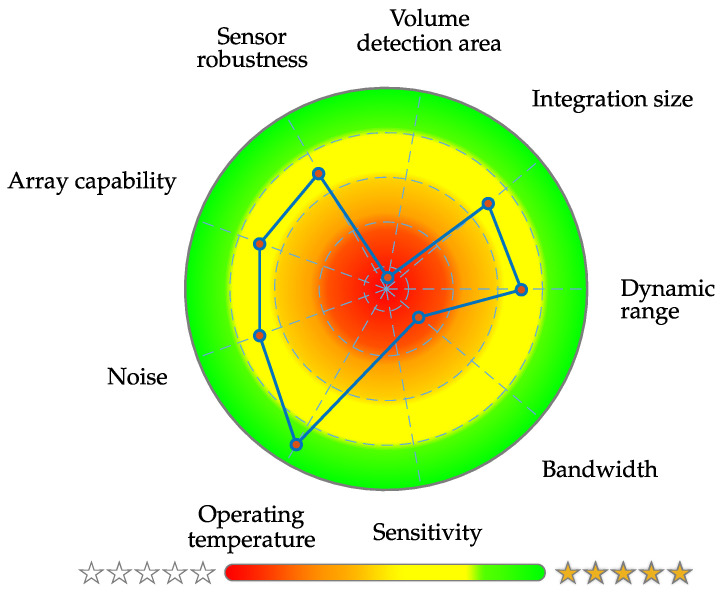
Exemplary prototype design for a clear presentation of application-related metrics [55].

The novel introduced *application-specific capacity* is a new figure of merit that utilizes details about the dominant frequencies of the desired signal and sensor noise. One weakness of the ASC is for sure the unfamiliar unit of dB Hz. The SNR definition is quite common and one can easily imagine how a signal with an SNR of 0 dB will roughly look like, because signal power and noise power will be in the same order of magnitude. An equivalent concern cannot be said about the ASC in the current state of research, because there is no intuition value mapping of how high the ASC needs to be for a sensor system to be suitable for a specific application. In addition, no data are yet available regarding the expressiveness of the ASC. There is an awareness of this issue and is planned to be addressed it in future research, which means a definition of application-depended prototype signals, noise quantification for various sensor types and an ASC mapping to qualitative measures. Finally a detailed signal evaluation should be performed with medical specialists of the specific field in an iterative fashion. In further investigations, a clear presentation of the individual system and application-related metrics is desirable, which can be achieved, for example, with a special type of pie chart. This diagram is called *target performance profile* and a first prototype design is exemplary shown in Figure 14. Nevertheless, further studies are necessary for an adequate metric normalization and comparability of the different magnetometers.

## 6. Conclusions

In this work, essential metrics for sensor system assessment were initially defined and complemented by a novel application-specific signal evaluation. Currently, such an ME sensor system evaluation approach is not available, and the literature and standardization so far can be described as inadequate. Table 2 presents exemplary essential ME system evaluation metrics and values for a quantitative signal evaluation concerning a cardiological application. Certainly, practical considerations, for example, readout method, integration size, volume of the detection area, array capability and robustness, can also be included in a further step. These quantities are not considered in this contribution since it concerns purely qualitative quantities, where a fundamental definition of a uniform ordinal scale is indispensable. This paper is meant as a starting point for the assessment of biomagnetic sensor systems by supporting the accurate differentiation and use of sensor system metrics like *Limit-of-Detection*, *noise* and *bandwidth*. Additionally, it shows the possibility for end-users, for example, medical doctors, to select the appropriate sensor system. Beyond that, this novel approach could help to quantify and subsequently optimize the system performance of already available magnetometers (e.g., alkali OPMs [56] or Helium OPMs [57]) as well as new ones (e.g., ultra-sensitive xMR-Sensors [58]) for a particular biomagnetic application. Thus, it will enable the benchmarking of individual systems for both single and multi-sensor approaches. In conclusion, our presented system evaluation can lead to an application-oriented system comparison. In addition to its potential support in sensor research and development, the quantitative metrics might prove helpful for performance optimization. On this basis, the usability of available and upcoming sensor systems can be easily verified for new applications.

## Figures and Tables

**Table 1:** Two researched ME sensors with their individual metrics given by publications.

Metrics	Exchange Bias ME Sensor [13]	Electrically Modulated ME Sensor [10,16]
Operation	Room	Room
Temperature	temperature	temperature
Inherent	≈4 pT/$\sqrt{\mathrm{Hz}}$	≈70 pT/$\sqrt{\mathrm{Hz}}$
Noise	at 7.684 kHz	at 10 Hz
Bandwidth	≈12.5 Hz (−6 dB)	unknown
Sensitivity	≈98 kV/T	≈40 kV/T
Availability	under development	under development

**Table 2:** Comparison of magnetic field sensor systems evaluated within this contribution.

Parameters	Exchange Bias ME Sensor	Electrically Modulated ME Sensor
**Amplitude Response**		
$f_{\mathrm{res}}$	7684 Hz	
$f_{-3\mathrm{dB}}$	$f_{-3\mathrm{dB},1}$ = 7680 Hz (low)$f_{-3\mathrm{dB},2}$ = 7689 Hz (high)	11.3 kHz
$f_{-6\mathrm{dB}}$	$f_{-6\mathrm{dB},1}$ = 7677 Hz (low) $f_{-6\mathrm{dB},2}$ = 7692 Hz (high)	15 kHz
*Q*	854	
$\left|{\mathrm{Slope}}_{-3\mathrm{dB}/-6\mathrm{dB}}\right|$	0.94 dB/Hz (low) 0.92 dB/Hz (high)	0.805 dB/kHz
$B_{3\mathrm{dB}}$	9 Hz (bandpass)	11.3 kHz (lowpass)
$B_{6\mathrm{dB}}$	15 Hz (bandpass)	15 kHz (lowpass)
**Sensitivity**		
$\epsilon_{\mathrm{sys}}$	63 kV/T at $f_{\mathrm{res}}$	5.76 kV/T at 10 Hz
**Noise**		
$\sqrt{{\hat{S}}_{B}\left(f\right)}$	6 pT/$\sqrt{\mathrm{Hz}}$ at $f_{\mathrm{res}}$	66 pT/$\sqrt{\mathrm{Hz}}$ at 10 Hz
$B_{n}^{\mathrm{rms}}$	20 pT ($f_{-3\mathrm{dB},\mathrm{low}}$ to $f_{-3\mathrm{dB},\mathrm{high}}$)	11.7 nT (1 Hz to $f_{-3\mathrm{dB}}$)
**Input-Output-Relation**		
${\bar{b}}_{\mathrm{out}}$	11 pT	55 pT
LOD	22 pT	102 pT
LOQ	42 pT	210 pT
$b_{1\mathrm{dB}}$	6 µT	18 µT
$b_{3\mathrm{dB}}$	18 µT	23 µT
$b_{\max}$		27 µT
DR	103 dB	98 dB
**Application (MCG) Specific Quantities**		
SNR	−90 dB	−11 dB
ASC	9.8 $\times 10^{-7}$ dB Hz	23 dB Hz

## Data Availability

Not applicable.

## Abbreviations

The following abbreviations are used in this manuscript:

A/D	Analog/Digital
ACF	Autocorrelation function
AS	Amplitude spectrum
ASC	Application specific capacity
ASD	Amplitude spectral density
DFT	Discrete Fourier transform
DR	Dynamic range
ECG	Electrocardiography
EEG	Electroencephalography
EMC	Electromagnetic compatibility
ENBW	Equivalent noise bandwidth
HP	Highpass
JFET	Junction-gate-field-effect transistor
LOD	Limit-of-Detection
LOQ	Limit-of-Quantification
LTI	Linear time-invariant
ME	Magnetoelectric
MCG	Magnetocardiography
OPM	Optically Pumped Magnetometer
PCB	Printed circuit board
PS	Power spectrum
PSD	Power spectral density
PTB	Physikalisch Technische Bundesanstalt
RMS	Root mean square
SNNR	Signal-plus-noise to noise ratio
SNR	Signal-to-noise ratio
SQUID	Super conducting quantum interference device

## Appendix A. MCG Prototype Signal

**Table A1:** Support points for MCG prototype signal.

*t* [s]	0	0.25	0.3	0.35	0.44	0.47	0.5	0.52	0.56	0.6	0.75	0.85	1
s(t) [pT]	0	0	2.1	0	0	−10.5	70	−7	0	0	12.6	0	0
$\frac{ds(t)}{dt}$ [$\frac{\mathrm{pT}}{s}$]	0	0	0	0	0	0	0	0	0	0	0	0	0
